# Associations with thrombosis are stronger for antiphosphatidylserine/prothrombin antibodies than for the Sydney criteria antiphospholipid antibody tests in SLE

**DOI:** 10.1177/09612033211014570

**Published:** 2021-05-06

**Authors:** Sahwa Elbagir, Giorgia Grosso, NasrEldeen A Mohammed, Amir I Elshafie, Elnour M Elagib, Agneta Zickert, Vivek Anand Manivel, Eleftheria Pertsinidou, Musa A. M Nur, Iva Gunnarsson, Johan Rönnelid, Elisabet Svenungsson

**Affiliations:** 1 Department of Immunology, Genetics and Pathology, Uppsala University, Uppsala, Sweden; 2 Division of Rheumatology, Department of Medicine Solna, Karolinska Institutet, Karolinska University Hospital, Stockholm, Sweden; 3 Faculty of Medical Laboratory Sciences, Al Neelain University, Khartoum, Sudan; 4 Department of Medical Biosciences, Pathology, Umeå University, Umeå, Sweden; 5 Rheumatology Unit, Military Hospital, Omdurman, Sudan; 6 Rheumatology Unit, Alribat University Hospital, Khartoum, Sudan

**Keywords:** Antiphosphatidylserine/prothrombin, carotid plaques, lupus anticoagulant, systemic lupus erythematosus, thrombosis

## Abstract

**Objectives:**

Antiphosphatidylserine/prothrombin complex antibodies (aPS/PT) are risk factors for thrombosis, yet further validation of their clinical relevance in different ethnic groups is required. We investigated the performance of aPS/PT of IgA/G/M isotypes among Sudanese and Swedish systemic lupus erythematosus (SLE) patients.

**Methods:**

Consecutive SLE patients/matched controls from Sudan (n = 91/102) and Sweden (n = 332/163) were included. All patients fulfilled the 1982 ACR SLE classification criteria. IgA/G/M of aPS/PT, anti-cardiolipin and anti-β_2_glycoprotein I (anti-β_2_GPI) were tested in both cohorts, and lupus anticoagulant (LA) also in the Swedish cohort. Clinical antiphospholipid syndrome-related events and atherosclerosis, measured as carotid plaques were assessed for associations. Univariate and multivariate analyses adjusting for cardiovascular risk factors were performed.

**Results:**

Sudanese SLE patients had higher levels of IgM aPS/PT, but using national cut-offs, the frequency of positivity was similar to Swedish patients for all isotypes. Among Swedish patients, all isotypes of aPS/PT associated with venous thromboembolism (VTE), while only IgA aPS/PT associated with arterial thrombosis (AT). aPS/PT antibodies associated strongly with LA and they were, independently, the best predictor for VTE. Double positivity for aPS/PT and anti-β_2_GPI associated with higher VTE risk than the conventional triple positivity. Carotid plaques did not associate with any antiphospholipid antibody.

**Conclusions:**

IgA aPS/PT associated with AT, and the association of IgG/M aPS/PT with VTE outperforms LA and criteria antiphospholipid antibodies in Swedish SLE patients. Furthermore, double positivity for aPS/PT and anti-β_2_GPI performed better than conventional triple positivity. Future studies need to address if aPS/PT can replace LA, as this would simplify clinical procedures.

## Introduction

Antiphospholipid antibodies (aPL) are directed against a wide range of phospholipids and phospholipid-binding plasma proteins, of which anti-β_2_ glycoprotein I (anti-β_2_GPI) and anti-cardiolipin (anti-CL) are known to be associated with thrombotic clinical events. IgG/M isotypes of anti-β_2_GPI, anti-CL, and/or lupus anticoagulant (LA) positivity, comprise the laboratory criteria for classification of the antiphospholipid syndrome (APS).^1^ Additionally, aPL of the immunoglobulin (Ig) A isotype is now included in the two most recent sets of classification criteria for systemic lupus erythematosus (SLE).^2,3^

Since the original description of APS,^4,5^ other antibodies targeting phospholipid and/or plasma proteins that associate with thrombotic events have been reported. Autoantibodies directed against the phosphatidylserine/prothrombin complex (aPS/PT) have raised mounting clinical interest in recent years.^6,7^ In the presence of calcium ions, prothrombin (factor II in the coagulation cascade) binds to negatively charged phospholipids e.g. phosphatidylserine through its vitamin K-dependent carboxylation domains.^8^ Associations with thrombotic and adverse pregnancy outcomes are more convincing for aPS/PT as compared to another set of antibodies which target prothrombin directly, not bound to negatively charged phosphatidylserine.^6,9,10^ A recent systematic review concluded that there is a strong evidence for positive associations between aPS/PT antibodies and APS manifestations, suggesting that these tests merit clinical use.^7^ Consequently, aPS/PT antibodies were also included in risk stratification scoring systems the antiphospholipid score (aPL-S)^11^ and the global APS score (GAPSS)^12^ to evaluate asymptomatic aPL positive patients. To what extent aPL contribute to accelerated atherosclerosis is debated, and no studies have investigated whether aPS/PT antibodies relate to measures of atherosclerosis.

Of the criteria APS tests, a positive LA has the strongest association with clinical APS manifestations.^13^ LA positivity has been demonstrated to be mainly dependent on β_2_GPI and PS/PT as antigens.^14–16^ Tetra-positivity including the three criteria tests plus aPS/PT was recently reported to impose even higher risk for APS-related events compared to the conventional triple positivity (anti-β_2_GPI, anti-CL and LA).^17,18^ Despite the documented high concordance between LA and aPS/PT,^16,19,20^ the latter was also shown to be a risk factor for thrombosis, independent of LA activity.^19^ Nevertheless, there is still not enough evidence to endorse the utility of aPS/PT as a substitute biomarker to LA.^21^ Further validation of the role of aPS/PT in clinical settings is even more essential as it might overcome the technical challenges of LA testing in different laboratories, and the corresponding uncertain results in patients on regular anticoagulant medications.^22,23^

To our knowledge aPS/PT have never been investigated in populations of African origin affected by SLE or APS. In the current study we include SLE patients from Sudan and Sweden and matched controls from both countries. Our aim was to evaluate the prevalence of aPS/PT, also including IgA isotypes. We assessed the clinical significance and compared the performance with the well-established criteria aPL, including LA activity.

## Methods

### Participants and clinical characteristics

Ninety-one Sudanese and 332 Swedish SLE patients, and 102 and 163 age- and sex-matched national controls were included in the study. All patients fulfilled the 1982 revised SLE criteria.^24^ Sudanese controls were healthy individuals; university staff and students and Swedish controls were population controls where SLE was the exclusion criterion. Detailed description of the cohorts was reported in our previous publication.^25^ Clinical information was obtained from patient records, interviews and clinical examination at time of inclusion: history of thrombotic events (venous thromboembolism (VTE): deep venous thrombosis confirmed by venography or ultrasonography or pulmonary embolism confirmed by radionuclide lung scanning or angiogram, and arterial thrombosis (AT): cerebrovascular events diagnosed by computer tomography or magnetic resonance imaging, myocardial infarction confirmed by electrocardiography and a rise in plasma creatine kinase-MB or Troponin T, or ischemic peripheral tissue loss), obstetric events (any history of recurrent early miscarriage, late miscarriage or intra-uterine fetal demise (IUFD)). Thrombocytopenia at study inclusion was recorded for Swedish patients, and history of immune thrombocytopenic purpura (ITP) for the Sudanese patients. Cardiovascular disease (CVD) risk factors included in the study were: age, gender, arterial hypertension (systolic and/or diastolic blood pressure ≥140/90 mmHg or use of antihypertensive medications), hyperlipidemia (low-density lipoprotein (LDL) > 3 mmol/L or treatment with lipid lowering agents) and smoking (never or ever smokers). As a measure of atherosclerosis, ultrasound scans were performed in 288 patients from Sweden using a duplex scanner with a linear array transducer. Scans were digitalized for off-line analysis. Pictures were frozen incident with the R-wave on the electrocardiogram. Carotid plaques were defined as a local increase in wall thickness of > 1 mm and >100% increase in wall thickness compared with adjacent wall. More methodological details have been previously published.^26^

All participants gave written informed consent and the study conformed to the guidelines of Declaration of Helsinki. The Ethics Committees of Alribat University hospital, Khartoum, Sudan, Omdurman Military hospital, Omdurman, Sudan (11 April 2011 and 25 May 2011, respectively) and the Uppsala and Karolinska University Hospital approved the study (03-556 [16 December 2003]).

### Antiphospholipid antibodies testing

Quantification of IgA/G/M aPS/PT, anti-CL and anti-β_2_GPI was performed using the Aptiva system based on a particle-based multi-analyte technology (PMAT) (Inova Diagnostics, San Diego, CA, USA; under development and for research use only^27^). Blood samples were obtained and prepared on the day of clinical examination at the time of study inclusion; sera from Sudanese and Swedish subjects were used for the analyses. The number of patients investigated for aPS/PT differed slightly between the isotypes for Swedish and Sudanese patients, the exact numbers are shown in Supplementary Table 2. Measurement data and clinical associations for anti-CL and anti-β_2_GPI for Sudanese and Swedish subjects were previously published,^26,28,29^ and only used in this study for multivariate and comparative statistics with aPS/PT in Swedish patients. Information about LA positivity was available for Swedish patients and was determined by the modified Dilute Russel Viper Venom Time method (dRVVT) (Biopool, Umeå, Sweden; using Bioclot LA). Twenty-four out of 61 LA positive patients were on warfarin treatment, 17 (70.8%) of which had confirmed positive LA test on more than one occasion.

Three cut-offs were defined: the manufacturers’ suggested cut-offs, and the 95th and 99th percentiles of the respective national controls. Non-parametric statistics were used to identify national cut-offs since all aPL levels were non-normally distributed. Clinical evaluation of aPL in Sudanese patients did not use the 99th national cut-offs, as the number of Sudanese controls was limited (n = 106). To determine the clinical associations among Swedish patients, the 99th national cut-offs were used in all univariate and multivariate regression analyses.

### Statistical analyses

For comparisons between quantitative variables, Mann–Whitney’s U test was used, whereas Chi2-test was used to test for differences between categorical variables with Fisher’s exact test applied when appropriate. Among Swedish patients, thrombotic events and LA activity were defined as dependent variables; univariate and multivariate logistic regressions including aPL positivity determined by the 99th national cut-off were used to calculate odd ratios (OR) and 95% confidence intervals (CI). Subsequently, stepwise forward regression analyses, including CVD risk factors and LA when significant and all aPL, were performed to determine independent predictors. For age, OR was calculated per change in regressor for 10 years. Multivariate analyses were not performed in the Sudanese patients due to the limited cohort size and small number of thrombotic events.

All statistical analyses including cutoff determination were conducted using JMP statistical software (SAS institute, Cary, NC, USA). P values <0.05 were considered significant.

## Results

### aPS/PT isotypes in Sudanese and Swedish patients

Demographic data and clinical information are included in Supplementary Table 1.

Levels of IgM aPS/PT antibodies were higher among Sudanese compared to Swedish patients, whereas no difference in levels of IgA/G isotypes was observed (Table 1). Using manufacturer’s cut-offs, positivity of IgA aPS/PT was less frequent among Sudanese patients. Levels of aPS/PT differed between Sudanese and Swedish controls. Whereas IgA and IgM aPS/PT were higher among Sudanese than among Swedish controls (medians 43 vs. 41 MFI; p = 0.0009 and 78 vs. 69 MFI; p < 0.0001 respectively) IgG aPS/PT levels were higher among the Swedish controls (medians 229 vs. 242 MFI; p = 0.0007). After adjustments to respective national cut-offs (both the 95th and 99th of matched national controls), Sudanese and Swedish patients did not differ in the prevalence of any aPS/PT isotype (Table 1).

**Table 1.** aPS/PT isotypes among Sudanese and Swedish patients.

	Sudann = 91	Swedenn = 332	P
IgA aPS/PT median/IQR	46/42–52	44/41–53	0.2
IgG aPS/PT median/IQR	249/235–266	251/230–290	0.6
IgM aPS/PT median/IQR	83.5/71–100	71/63–95	**0.0002**
IgA aPS/PT manufacturer’s cutoff (>65 MFI)	3(3.3)	46(13.9)	**0.005**
IgG aPS/PT manufacturer’s cutoff (>300 MFI)	11(12.1)	68(20.9)	0.06
IgM aPS/PT manufacturer’s cutoff (>100 MFI)	20(22.7)	70(21.4)	0.9
IgA aPS/PT 95th cutoff (Sudan: >56 MFI, Sweden: >56 MFI)	13(14.3)	66(19.9)	0.2
IgG aPS/PT 95th cutoff (Sudan: >283 MFI, Sweden: >310 MFI)	14(15.4)	64(19.6)	0.3
IgM aPS/PT 95th cutoff (Sudan: >112 MFI, Sweden: >106 MFI)	17(19.3)	67(20.5)	0.8
IgA aPS/PT 99th cutoff (Sudan: >84 MFI, Sweden: >107 MFI)	2(2.2)	20(6)	0.1
IgG aPS/PT 99th cutoff (Sudan: >365 MFI, Sweden: >521 MFI)	3(3.3)	24(7.4)	0.2
IgM aPS/PT 99th cutoff (Sudan: >156 MFI, Sweden: >322 MFI)	7(8)	24(7.4)	0.8

Values are (median (MFI)/IQR) for continuous variables and n (%) for categorical data. Prevalence of antibodies was determined using three cutoffs: manufacturers’ cutoffs and separate cutoffs based on the 95th and 99th percentiles among national controls. Significant p values are depicted in bold.

aPS/PT: anti-phosphatidylserine/prothrombin; IQR: interquartile range; MFI: median fluorescent intensity.

Among Sudanese patients no associations of aPS/PT to venous or arterial thrombosis were found (Supplementary Table 2). Among Swedish patients, using both the 95th and 99th national cut-offs, VTE associated with all aPS/PT isotypes, whereas only IgA aPS/PT associated with AT. IgG aPS/PT associated with thrombocytopenia both in Sudanese (the 95th cut-off) and Swedish SLE patients (the 95th and 99th cut-offs). aPS/PT did not associate with obstetric complications in any cohort.

### Extended analyses of thrombotic events, aPL and LA in Swedish SLE patients

**Figure 1.**
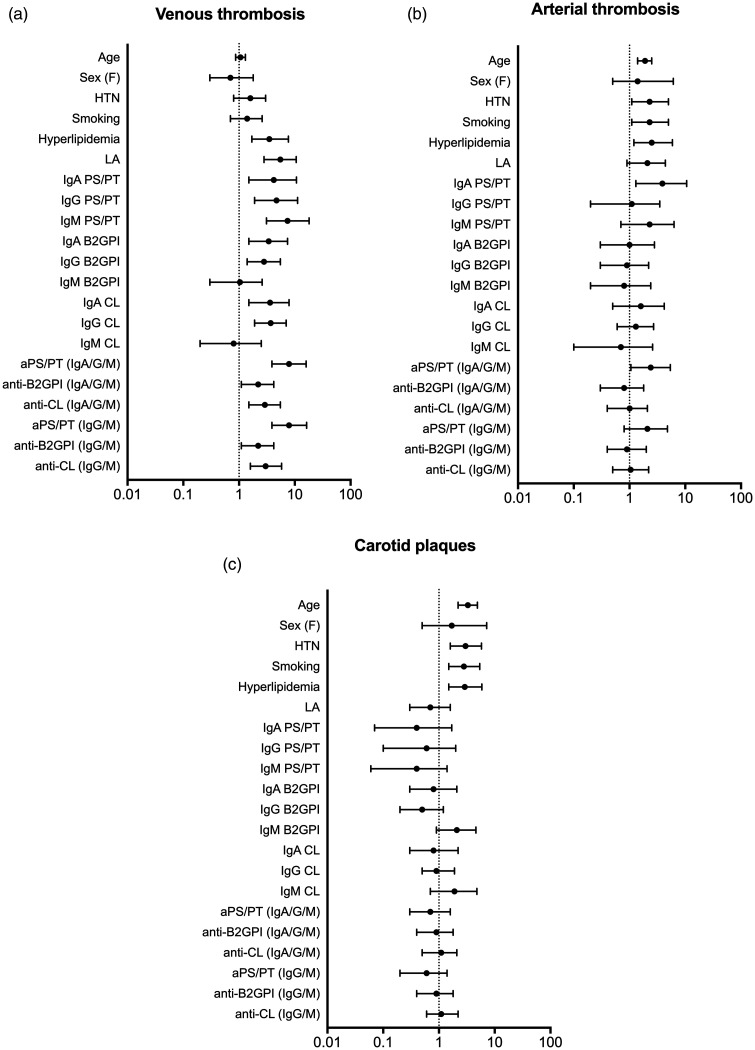
Associations of antiphospholipid antibodies (aPL) and cardiovascular risk factors with thrombosis and atherosclerosis in Swedish SLE patients. Odds ratios with confidence intervals for the occurrence of venous thrombosis (a), arterial thrombosis (b) and carotid plaques (c) are demonstrated. Univariate regression models were used to calculate odds ratios; for age it was calculated for 10-year change in regressor. Positivity for all aPL including aPS/PT was determined by the respective 99th national cut-off values. *y-*axis indicates independent variables while the *x*-axis shows odds ratios. F: female; HTN: arterial hypertension; LA: lupus anti-coagulant.

Univariate analysis of conventional CVD risk factors and different aPL isotypes (using the 99th cut-off), with vascular thromboses and carotid plaques as outcomes are shown in Figure 1. Hyperlipidemia, LA, all isotypes for aPS/PT but only IgA/G anti-β_2_GPI and IgA/G anti-CL significantly associated with VTE; the highest OR appeared for IgM aPS/PT (OR 7.4 [CI 3.1–18.1]). AT associated with older age, hypertension, smoking and hyperlipidemia, but most strongly with occurrence of IgA aPS/PT (OR 3.9[CI 1.3–10.6]). aPS/PT did not associate with history of myocardial infarction or the presence of carotid plaques, however IgA aPS/PT, LA and older age associated with cerebrovascular events.

#### Multivariate associations with vascular events

**Figure 2.**
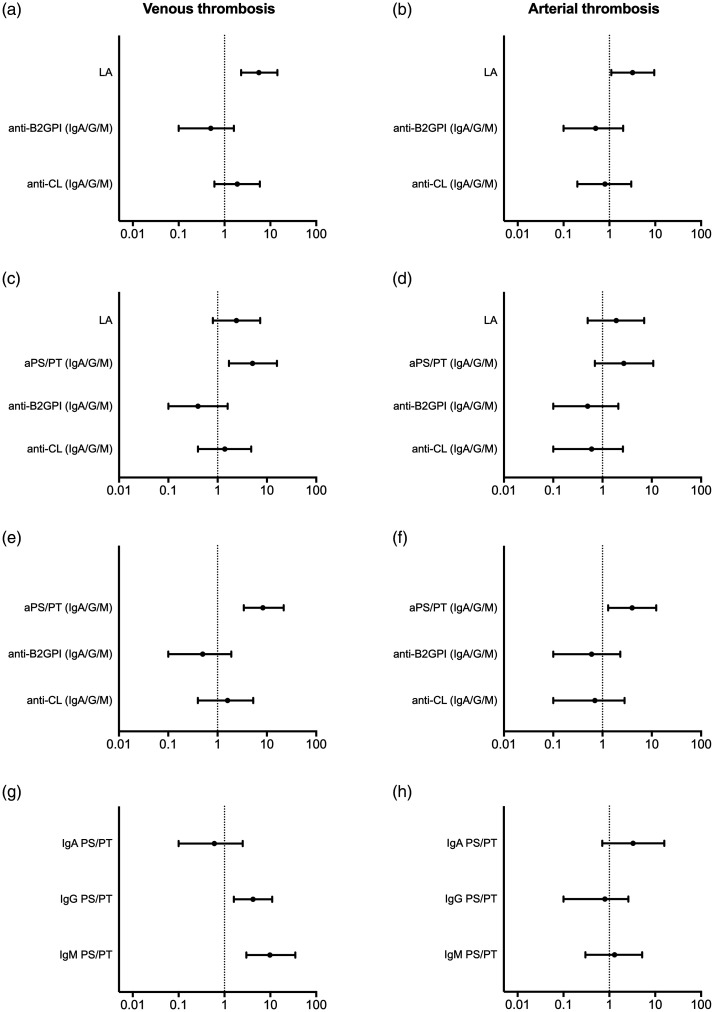
Associations of antiphospholipid antibodies (aPL) and lupus anticoagulant (LA) with thrombosis in Swedish SLE patients. Criteria APS tests are demonstrated as explanatory variable and venous (a) and arterial thrombosis (b) as outcomes. The effect of adding anti-phosphatidylserine/prothrombin (PS/PT) predicting venous (c) and arterial thrombosis (d) is shown, and thereafter with exclusion of LA in (e) and (f). The associations of individual aPS/PT isotypes to venous (g) and arterial thrombosis (h) are also demonstrated. Positivity for all aPL including aPS/PT was determined by the respective 99th national cut-off values. *y-*axis indicates independent variables while the *x*-axis shows odds ratios. Multiple logistic regressions were used to calculate all odds ratios.

In a multiple logistic regression model including the three APS criteria tests, LA positivity was the only independent risk factor for VTE and AT (Figure 2(a) and (b)). However, when we added aPS/PT antibodies to that model, only aPS/PT and not LA was an independent risk factor for VTE (Figure 2(c)), and no associations were significant for AT (Figure 2(d)). In a multiple regression model including aPS/PT, anti-β_2_GPI and anti-CL (any isotype), only aPS/PT positivity was a significant risk factor for thrombosis (Figure 2(e) and (f)). Subsequently when combining only the three aPS/PT isotypes in the analysis, IgG and IgM associated with VTE while IgA aPS/PT lost significance for AT (Figure 2(g) and (h)). Stepwise regression including statistically significant CVD risk factors from the univariate analyses, LA and all aPL specificities revealed significant association of VTE with IgM aPS/PT, LA and hyperlipidemia. In a similar model, AT associated with IgA aPS/PT and older age (data not shown). IgA aPS/PT and age were also independently associated with cerebrovascular events (OR 5.1 [CI 1.3–16.8] and OR 1.6 [CI 1.1–2.2]), respectively.

#### Predictors of LA positivity

**Figure 3.**
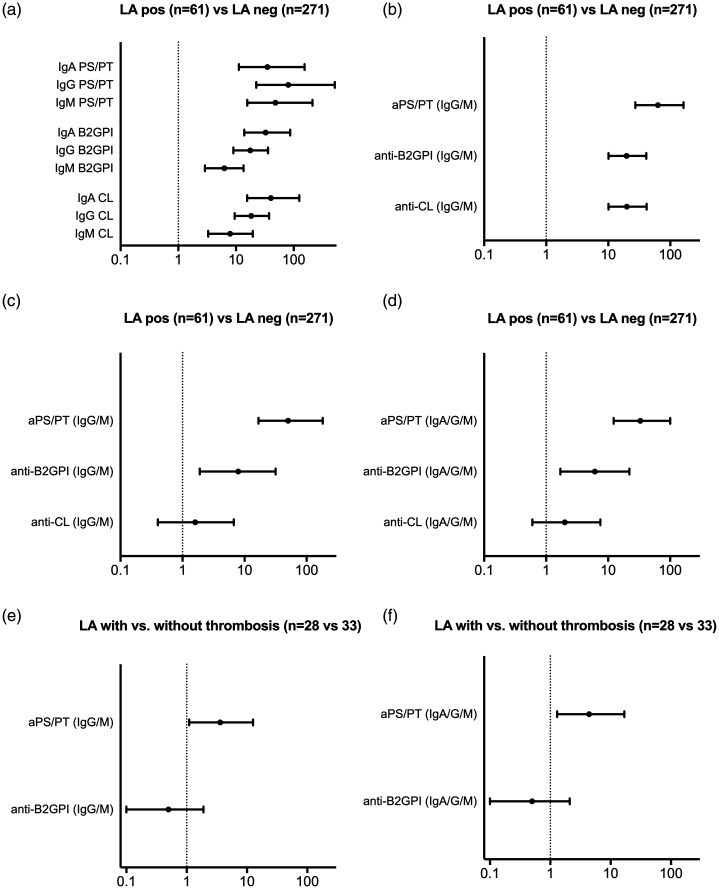
Predictors for a positive lupus anticoagulant (LA) test in Swedish SLE patients. Using univariate statistics, odds ratios of the individual antiphospholipid antibodies (aPL) (a) and any IgG/M aPL (b) are demonstrated. Using multiple regressions, the association between any IgG/M (c) and IgA/G/M (d) of aPS/PT, anti-β_2_GPI and anti-CL with LA and the association of IgG/M (e) and IgA/G/M (f) of aPS/PT and anti-β_2_GPI with LA in patients with thrombosis vs. without thrombosis are demonstrated. Positivity for all aPL including aPS/PT was determined by the respective 99th national cut-off values. *y-*axis indicates independent variables while the *x*-axis shows odds ratios.

Sixty-one out of 332 Swedish patients were LA positive (18.4%). All aPL predicted LA positivity in univariate analysis, with IgG/M aPS/PT showing the strongest associations (Figure 3(a) and (b)). Using multiple logistic regression, including the three aPL specificities, both aPS/PT and anti-β_2_GPI positivity predicted LA positivity (Figure 3(c) and (d)). However, among the LA positive patients, only aPS/PT associated with previous thrombotic incidents (Figure 3(e) and (f)).

When we excluded LA positive patients treated with warfarin (n = 24), similar results were obtained regarding associations of aPL with LA activity; both aPS/PT and anti-β_2_GPI predicted LA in univariate and multivariate models (Supplementary Figure 1a–d). However, due to the limited number of patients with LA activity and thrombotic events (n = 8) we could not perform regression models (as done in Figure 3(e) and (f)), in order to investigate the associations of aPS/PT and anti-β_2_GPI antibodies to pathogenic LA. Also, after exclusion of the warfarin-treated patients the association between LA and venous thrombosis was lost.

#### Triple positivity

Interestingly, simultaneous positivity for anti-β_2_GPI (IgG or IgM), aPS/PT (IgG or IgM) and LA conferred higher risk for VTE compared to conventional triple positivity (IgG/M anti-β_2_GPI and IgG/M anti-CL and LA)^30^ (OR 7.1[CI 3.1–16] vs. OR 5.2[CI 2.5–10.7]) (Table 2). Also, double positivity for IgG/M anti-β_2_GPI and IgG/M aPS/PT was associated with higher VTE risk (OR 6.3[CI 2.8–13.9]), compared to the conventional triple positivity.

**Table 2.** Thrombotic risk of double and triple positivity of the different anti-phospholipid tests.

	OR (CI) VTE	OR (CI) AT
Triple positivity (IgG/M anti-β2GPI, anti-CL and LA)	5.2 (2.5–10.7)	1.5 (0.5–3.7)
Triple positivity (IgG/M anti-β2GPI, aPS/PT and LA)	7.1 (3.1–16.0)	1.3 (0.4–3.7)
Double positivity (IgG/M anti-β2GPI and aPS/PT)	6.3 (2.8–13.9)	1.6 (0.5–4.2)
Triple positivity (IgA/G/M anti-β2GPI, anti-CL and LA)	5.0 (2.4–10.2)	1.5 (0.5–3.6)
Triple positivity (IgA/G/M anti-β2GPI, aPS/PT and LA)	8.1 (3.7–17.8)	1.2 (0.3–3.3)
Double positivity (IgA/G/M anti-β2GPI and aPS/PT)	6.8 (3.1–14.5)	1.8 (0.6–4.4)

Positivity for all aPL including anti-PS/PT was determined by the respective 99th national cut-off values.

aPS/PT: anti-phosphatidylserine/prothrombin; AT: arterial thrombosis; β2GPI: β2 glycoprotein I; CI: confidence interval; CL: cardiolipin; LA: lupus anticoagulant; OR: odd ratio; VTE: venous thromboembolism.

#### Co-occurrence of aPL in Swedish SLE patients

**Figure 4.**
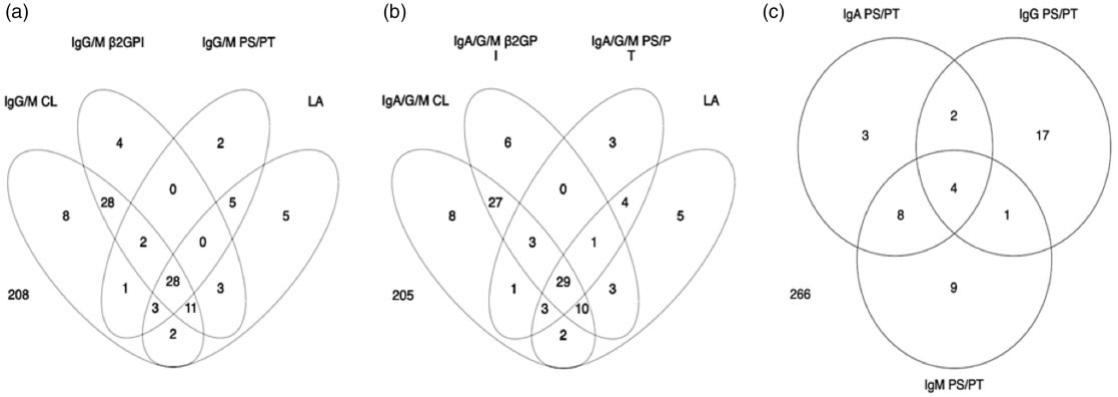
Venn diagrams of the different antiphospholipid isotypes and lupus anticoagulant among the Swedish SLE patients. Occurrence of IgG/M (a) and IgA/G/M (b) of aPS/PT, anti-β_2_GPI and anti-CL and LA, and in (c) the different isotypes of aPS/PT antibodies, are shown. Positivity for all aPL including aPS/PT was determined by the respective 99th national cut-off values. Patients with full data on all measures (n = 310) were included.

aPL positive patients mostly fell into two main clusters; a cluster with quadruple positivity for anti-CL, anti-β_2_GPI, aPS/PT and LA, and another with double positivity for anti-CL and anti-β_2_GPI where LA and aPS/PT were negative. IgA aPS/PT commonly co-occurred with IgG/M aPS/PT (Figure 4).

## Discussion

In this study IgM/G aPS/PT antibodies were stronger risk factors for VTE than LA and conventional aPL. For arterial events IgA aPS/PT was the only test to have a significant positive association, thus outperforming all the “criteria APS tests”. When combining test results, we noted that double positivity for any isotype of anti-β_2_GPI and any isotype of aPS/PT performed better than triple positivity by the conventional markers anti-CL, anti-β_2_GPI and LA. We also confirm that LA is more strongly associated with aPS/PT than with anti-β_2_GPI.

Previous studies have demonstrated associations of both IgG and IgM aPS/PT to APS-related events,^6,7^ but only few researchers reported multivariate adjustments for the criteria aPL^31,32^ and/or conventional thrombotic risk factors.^20,33^ In the current study, occurrence of IgM aPS/PT was the strongest risk factor for VTE in univariable analysis of Swedish SLE patients. This association remained independent of the conventional aPL and CVD risk factors. This is an interesting observation, since the association between thrombotic events and IgM isotypes of anti-CL/anti-β_2_GPI have been questioned,^33,34^ a view which is supported by our results as we did not see any associations with arterial or venous thrombosis.

In clinical practice VTE and AT are treated as separate entities as they are known to differ regarding risk profiles and treatments. Consequently, we analyzed risk factors for VTE and AT separately, though a considerable number of previous aPL studies combined VTE and AT to a composite thrombotic outcome.^35,36^ We noted that hyperlipidemia, defined by high LDL or lipid lowering treatment, was independently associated with VTE in Swedish SLE patients. In some former reports, positive associations between VTE and dyslipidemias were also reported,^37^ though in general hyperlipidemia is a well-known risk factor for arterial, and not venous events.^38^ Our observations might be true, but can also reflect confounding by other factors that were not adjusted e.g., obesity and renal disease.

Published data on the role of aPS/PT as a risk factor specifically for AT are inconsistent; some studies have demonstrated positive associations,^32,39,40^ whereas others did not.^20,41^ Zigon et al. recently described a positive association between IgA/G aPS/PT and thrombosis defined as a combination of venous and arterial events.^33^

In our study, IgA aPS/PT was the strongest risk factor for AT and to our best knowledge we are the first group to report this association using both uni- and multivariate statistical methods including criteria aPL and CVD risk factors. Murthy et al previously reported that in SLE patients isolated IgA anti-β_2_GPI positivity remained as an independent risk factor for AT but not VTE, after adjustment for other risk factors.^42^ Furthermore, we recently reported similar exclusive association of IgA anti-β_2_GPI domain1 antibodies.^29^ In our study IgA aPS/PT commonly co-occurred with IgG/M aPS/PT isotypes. Further studies are clearly needed to investigate triggers, the role and temporal patterns of different aPL isotypes.

While the link between aPL and vascular events is well established, associations between aPL and measures of atherosclerosis is debated. We did not observe any associations between carotid plaques, a robust measure of subclinical atherosclerosis, and aPL of any specificity in this study. Similar observations were reported in prospective studies by Mc Mahon M et al.^43^ and Kiani AN et al.,^44^ whereas Kravvariti et al., who investigated the combined plaque occurrence in carotid and femoral arteries, found positive associations with a combination of criteria aPL.^45^ To understand if aPL contribute to the development of premature atherosclerosis needs to be investigated further.

LA positivity was better predicted by aPS/PT than by anti-β_2_GPI in the Swedish SLE patients, and only anti-PS/PT significantly correlated with pathogenic LA, defined as LA associated with thrombotic events. While aPS/PT independently associated with VTE, LA positivity lost this association when we adjusted for the presence of these antibodies. These data strengthen the narrative that aPS/PT could be a surrogate biomarker for LA, or even a more specific biomarker to identify the LA positive subgroup with high risk of thromboses. In a former study Shi H et al. investigated Chinese patients with primary and secondary APS, describing aPS/PT as a superior predictor of thrombosis compared to LA.^46^ Notably, the point estimate for VTE in Swedish SLE patients was higher for double positivity for aPS/PT together with anti-β_2_GPI antibodies than for conventional triple positivity further supporting a pivotal role of aPS/PT antibodies.

The LA test is poorly standardized and it is also often missed in clinical practice due to practical problems, as it requires fresh or fresh frozen citrate plasma.^22^ In the clinic it would be advantageous if aPS/PT can replace or serve as a surrogate measure of LA when the latter is technically unfeasible.

As there are no previous studies on the prevalence and role of aPS/PT in patients from the African continent, our aim of investigating the Sudanese subjects was to look for possible clinical associations and compare with Swedish patients. This intention was also driven by the clear differences in conventional aPL that we had observed between Sudanese and Swedish SLE patients in our previous publication.^29^ Although the presence of aPS/PT in Sudanese SLE patients did not associate with thromboses, we speculate that this statistical insignificance is a result of the low number of events in this patient group.

Main limitations of this study are the retrospective design and the low number of patients from Sudan, where we were not able to perform the LA test and where we could not collect as detailed clinical information as in the Swedish cohort. In addition, we did not adjust for ongoing treatment e.g. hydroxychloroquine and steroids as clinical events were collected retrospectively. A prospective study with expansion of the Swedish SLE cohort is now being performed. An interesting question to also investigate is whether levels and performance of aPS/PT antibodies as risk predictors are affected by SLE treatment e.g. hydroxychloroquine.

To our best knowledge, this is the first study to demonstrate the association between IgA aPS/PT antibodies and arterial events in SLE patients. More importantly, double positivity for aPS/PT and anti-β_2_GPI tests conferred higher VTE risk than the conventional triple positivity, a finding that may simplify aPL testing in the clinic. Further prospective studies are needed, and it is especially important to explore IgA aPS/PT as a risk factor for vascular outcomes.

## Supplemental Material

sj-pdf-1-lup-10.1177_09612033211014570 - Supplemental material for Associations with thrombosis are stronger for antiphosphatidylserine/prothrombin antibodies than for the Sydney criteria antiphospholipid antibody tests in SLE Click here for additional data file. Supplemental material, sj-pdf-1-lup-10.1177_09612033211014570 for Associations with thrombosis are stronger for antiphosphatidylserine/prothrombin antibodies than for the Sydney criteria antiphospholipid antibody tests in SLE by Sahwa Elbagir, Giorgia Grosso, NasrEldeen A Mohammed, Amir I Elshafie, Elnour M Elagib, Agneta Zickert, Vivek Anand Manivel, Eleftheria Pertsinidou, Musa A. M Nur, Iva Gunnarsson, Johan Rönnelid and Elisabet Svenungsson in Lupus

sj-pdf-2-lup-10.1177_09612033211014570 - Supplemental material for Associations with thrombosis are stronger for antiphosphatidylserine/prothrombin antibodies than for the Sydney criteria antiphospholipid antibody tests in SLE Click here for additional data file. Supplemental material, sj-pdf-2-lup-10.1177_09612033211014570 for Associations with thrombosis are stronger for antiphosphatidylserine/prothrombin antibodies than for the Sydney criteria antiphospholipid antibody tests in SLE by Sahwa Elbagir, Giorgia Grosso, NasrEldeen A Mohammed, Amir I Elshafie, Elnour M Elagib, Agneta Zickert, Vivek Anand Manivel, Eleftheria Pertsinidou, Musa A. M Nur, Iva Gunnarsson, Johan Rönnelid and Elisabet Svenungsson in Lupus
