# Supplementary material for: Associations with thrombosis are stronger for antiphosphatidylserine/prothrombin antibodies than for the Sydney criteria antiphospholipid antibody tests in SLE
Source: Lupus. 2021 May 6;30(8):1289–99. doi: 10.1177/09612033211014570 (PMC8209767; doi:10.1177/09612033211014570)
Supplement: sj-pdf-1-lup-10.1177_09612033211014570 - Supplemental material for Associations with thrombosis are stronger for antiphosphatidylserine/prothrombin antibodies than for the Sydney criteria antiphospholipid antibody tests in SLE [file sj-pdf-1-lup-10.1177_09612033211014570.pdf]

Supplementary Table 1 Demographics and clinical events in the investigated SLE patients.

	Sudan n=91	Sweden n=332	P
Gender (F/M)	88/3	290/39	**0.02**
Age at inclusion (median/mean) Years	35/35.9	47.7/46.8	**<0.0001**
Disease duration (median/mean) years	4/4.8	14/17.7	**<0.0001**
Arterial hypertension	23 (25.3%)	120 (39.5%)	**0.01**
Smoking	3 (3.3%)	175 (53.2%)	**<0.0001**
Hyperlipidemia	NA	187 (57%)	NA
Carotid plaques	NA	56 (19.4%)	NA
Any thrombosis	10(11%)	76(23.5%)	**0.009**
Venous thrombosis	4(4.4%)	50(15.4%)	**0.006**
DVT	3 (3.3%)	43 (13.1%)	**0.008**
PE	1 (1.1%)	17 (5.2%)	0.5
Arterial thrombosis	7(7.7%)	36(11.1%)	0.3
Cerebrovascular	3 (3.3%)	21 (6.4%)	0.3
MI	1 (1.1%)	14 (4.2%)	0.1
Peripheral tissue loss	3 (3.3%)	5 (1.5%)	0.2
Recurrent early miscarriage	4(3.6%)	6(2.1%)	0.4
Late miscarriage	15(17%)	36(13.1%)	0.3
Intrauterine fetal demise	4(4.6%)	7(2.8%)	0.4
Thrombocytopenia	10 (11.1%)	8 (2.3%)	NA

Abbreviations: DVT, deep venous thrombosis; F, female; M, male; MI, myocardial infarction; NA, not applicable; PE, pulmonary embolism.

Note: The occurrence of thrombocytopenia was documented at different time points for Sudan and Sweden, therefore was not statistically compared between the countries. Information for lipid indices and carotid plaques were not available for Sudanese patients.

Supplementary Table 2 aPS/PT associations to clinical events in 91 Sudanese and 332 Swedish SLE patients.

**Sudan**	Any thrombotic Sudan=10 Sweden=76	P	Venous thrombosis Sudan=4 Sweden=50	P	Arterial thrombosis Sudan=7 Sweden=36	P	Any obstetric event Sudan=21 Sweden=42	P	Thrombocytopenia Sudan=10 Sweden=8	P
IgA aPS/PT 95th cutoff (n=91)	7.7%/11.5%	0.7	7.7%/3.8%	0.5	7.7%/7.7%	1	30.8%/23%	0.5	15.4%/10.4%	0.6
IgG aPS/PT 95th cutoff (n=91)	7.1%/11.7%	0.6	0/5.3%	1	7.1%/8%	1	23.1%/24.3%	0.9	38.5%/7.8%	**0.002**
IgM aPS/PT 95th cutoff (n=88)	11.8%/9.9%	1	11.8%/1.4%	0.09	5.9%/8.7%	1	25%/25%	1	18.7%/11.3%	0.4
**Sweden**										
IgA aPS/PT 95th cutoff (n=332)	40.9%/19%	**0.0002**	28.8%/12%	**0.0007**	18.2%/9.3%	**0.04**	16%/16.6%	0.9	4.5%/1.9%	0.2
IgG aPS/PT 95th cutoff (n=326)	43.7%/18.5%	**<0.0001**	34.4%/10.6%	**<0.0001**	15.6%/10.2%	0.2	23.9%/15.1%	0.1	7.8%/1.2%	**0.009**
IgM aPS/PT 95th cutoff (n=327)	30.3%/21%	0.1	25.8%/11.8%	**0.004**	10.6%/11%	0.9	8.3%/18.6%	0.08	3%/2.3%	0.7
IgA aPS/PT 99th cutoff (n=332)	60%/21%	**<0.0001**	40%/13.8%	**0.002**	30%/9.8%	**0.005**	7.1%/17%	0.3	5%/2.3%	0.4
IgG aPS/PT 99th cutoff (n=326)	50%/21.4%	**0.001**	41.7%/13.2%	**0.0002**	12.5%/11.2%	0.8	31.6%/15.5%	0.07	16.7%/1.3%	**<0.0001**
IgM aPS/PT 99th cutoff (n=327)	58.3%/20%	**<0.0001**	50%/11.8%	**<0.0001**	20.8%/10.1%	0.1	11.1%/17.1%	0.5	4.1%/2.3%	0.5

Abbreviations: aPS/PT, anti-phosphatidylserine/prothrombin.

Note: For Sudanese patients the 95th national cutoffs were used and for Swedes both the 95th and the 99th national cutoffs are shown. Data are demonstrated as percentage of patients with events among antibody positive patients (%)/ patients with events among antibody negative patients (%). Number of patients investigated for aPS/PT differed slightly for Swedes and is given in the left column. Significant p values are depicted in bold
