# Supplementary figures and images for: Associations with thrombosis are stronger for antiphosphatidylserine/prothrombin antibodies than for the Sydney criteria antiphospholipid antibody tests in SLE
Source: Lupus. 2021 May 6;30(8):1289–99. doi: 10.1177/09612033211014570 (PMC8209767; doi:10.1177/09612033211014570)

LA pos (n=37) vs LA neg (n=271)

A.

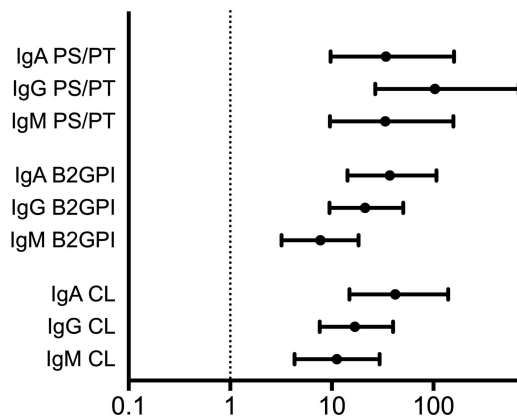

LA pos (n=37) vs LA neg (n=271)

B.

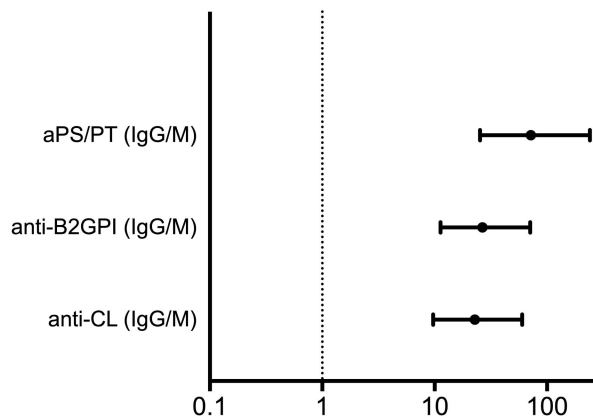

LA pos (n=37) vs LA neg (n=271)

C.

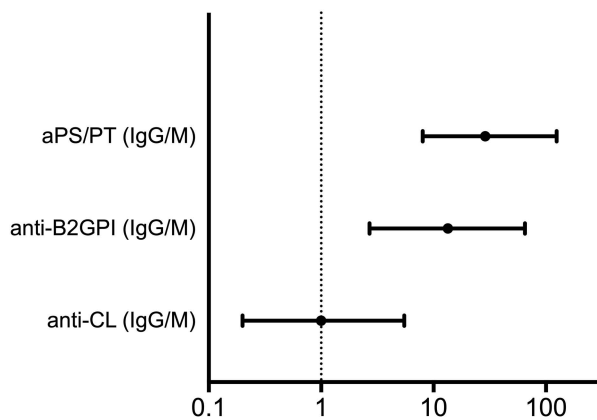

LA pos (n=37) vs LA neg (n=271)

D.

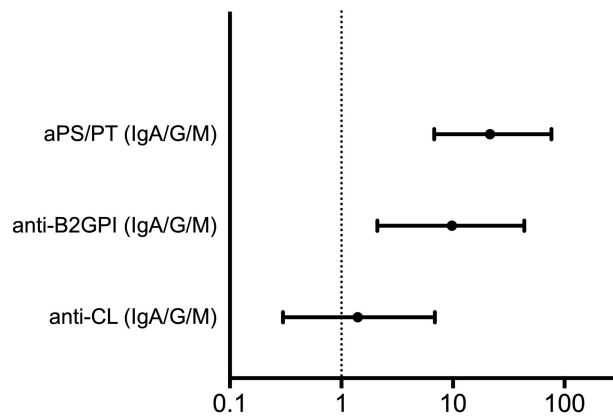

Supplement: sj-pdf-2-lup-10.1177_09612033211014570 - Supplemental material for Associations with thrombosis are stronger for antiphosphatidylserine/prothrombin antibodies than for the Sydney criteria antiphospholipid antibody tests in SLE [file sj-pdf-2-lup-10.1177_09612033211014570.pdf]
